# A systematic review of surgical procedures on thoracic myelopathy

**DOI:** 10.1186/s13018-020-02081-y

**Published:** 2020-12-10

**Authors:** Shiqi Zhu, Yu Wang, Peng Yin, Qingjun Su

**Affiliations:** Departmen of Orthopedics, Capital Medical University Affiliated Beijing Chaoyang Hospital, Capital Medical University, NO.8 Gongtinanlu, Beijing, 100020 People’s Republic of China

**Keywords:** Thoracic spine stenosis (TSS), Thoracic myelopathy (TM), Ossification of posterior longitudinal ligament (OPLL), Ossification of ligamentum flavum (OLF), Thoracic spine decompression

## Abstract

**Purpose:**

The surgical treatment of thoracic myelopathy is still controversial and also a challenge for spine surgeons. Therefore, the objective of this study was to review the related literature on the surgical treatment of thoracic myelopathy and try to define treatment guidelines for spine surgeons on thoracic myelopathy.

**Methods:**

Relevant literatures were searched based on the PubMed, EMBASE, and Cochrane Library between January 2008 and December 2018. Some data on the characteristics of patients were extracted, including number of patients, mean age, surgical procedures, blood loss, complications, and pre-/post-operation modified JOA score. Recovery rate was used to assess the effect of surgery outcome, and the safety was evaluated by blood loss and incidence of complications.

**Results:**

Thirty-five studies met the inclusion criteria and were retrieved. A total of 2183 patients were included in our systematic review, with the average age of 55.2 years. There were 69.8% patients diagnosed as ossification of ligamentum flavum (OLF), 20.0% as ossification of posterior longitudinal ligament (OPLL), 9.3% as disk herniation (DH), and 0.9% as others including diffuse idiopathic skeletal hyperostosis (DISH) and ankylosing spondylitis (AS). The volume of blood loss was more in the treatment of circumferential decompression (CD) than posterior decompression (PD), and the incidence of complications was higher in CD (*P* < 0.05). The volume of blood loss in minimally invasive surgery (MIS) was lowest and the incidence of complications was 19.2%. Post-operation recovery rate was 0.49 in PD, 0.35 in CD, and 0.29 in MIS while the recovery rate was 0.54 in PD, 0.55 in CD, and 0.49 in MIS at the last follow-up. When focusing on the OLF specifically, incidence of complications in PD was much lower than CD, with less blood loss and higher recovery rate. Focusing on the OPLL specifically, incidence of complications in PD was much lower than CD, with less blood loss while there was no statistical difference in recovery rate between these two methods.

**Conclusions:**

This systematic review showed that posterior decompression for thoracic myelopathy is safer and better than circumferential decompression according to the complication rate and surgical outcome. And we should also consider the location of compression before the operation.

## Background

The incidence of thoracic myelopathy is relatively lower among spine myelopathy, which is mainly caused by DH, OPLL, OLF, and others (DISH and AS). It has been reported that thoracic spine stenosis due to OPLL or OLF is the major etiology of thoracic myelopathy [1].

Patients with thoracic myelopathy always company with the symptoms of motor and sensory function reduction of lower limbs, and these symptoms often could not be relieved by conservative treatments, so surgical treatment gradually become the standard treatment for patients with thoracic myelopathy. However, the optimal surgical procedure is still controversial [2]. Posterior decompression and circumferential decompression through posterior approach were mostly applied on thoracic spine surgery, because nearby structures, such as aorta and esophagus, may be injured or damaged by anterior approach, and then the fatal complications might be encountered [3]. In recent years, minimally invasive surgery was preferred by some surgeons on the treatment of single lesion type of thoracic myelopathy, but the technique of the surgery was demanding [4, 5]. Although there are many surgical options up to now, there has not been a guideline or review to assist surgeons to choose a proper surgical approach for specific patient yet because of the complex etiologies and challenging surgical approach. Hence, in our following systematic review, we gathered the characteristics data of patients with thoracic myelopathy and compared the effect and safety of outcomes among these three surgical procedures (posterior decompressive, circumferential decompression through posterior approach, and minimally invasive surgery) and tried to give a valuable reference for surgeons to choose the optimal treatment for each patient with thoracic myelopathy.

## Methods

### Search strategy

Relevant literature searches were performed via PubMed, EMBASE, and Cochrane Library between January 1, 2008, and December 31, 2018. Keywords used to identify relevant studies were “thoracic spine stenosis,” “thoracic myelopathy,” “ossification of posterior longitudinal ligament,” and “ossification of ligamentum flavum.”

### Eligibility criteria

Eligibility criteria for article selection were as follows: (1) population: 10 more patients with thoracic myelopathy; (2) intervention: posterior decompression, circumferential decompression, or minimally invasive surgery; (3) outcome: motor and sensory function evaluated by modified JOA score; (4) study type: prospective or retrospective case series; and (5) full-text published articles between January 1, 2008, and December 31, 2018, and available in English.

### Date collection

All relevant data that met eligibility criteria were independently extracted by two authors, and the disagreements were resolved by discussion with each other. The following information was extracted from each article: (1) first author, (2) publish year, (3) patient’s demographics, (4) characteristics of thoracic myelopathy, (5) surgical approach, (6) preoperative and postoperative modified JOA score (mJOA), and (7) complications. Modified Japanese Orthopedic Association Scoring System (mJOA score) were applied to assess the motor and sensory function of patient and recovery rate (RR) for surgical outcomes. The recovery rate was calculated as:

$$ \mathrm{RR}=\left(\mathrm{postoperative}\ \mathrm{mJOA}\ \mathrm{score}-\mathrm{preoperative}\ \mathrm{mJOA}\ \mathrm{score}\right)/\left(11-\mathrm{preoperative}\ \mathrm{mJOA}\ \mathrm{score}\right)\ast \times 100\% $$

There were amount of surgical procedures used to decompress the thoracic spine cord mostly through posterior approach. Therefore, we categorized the different techniques into three major procedures: (1) posterior decompression (PD); (2) circumferential decompression (CD), vertebra fused or not; and (3) minimally invasive surgery (MIS) where decompression of thoracic spine was given through a posterior minimally invasive way. This nomenclature was based on the differences in decompression mechanisms among the various techniques.

### Statistical analysis

The statistical differences among surgical procedures were analyzed by chi-square test and *t* test, and the statistical significance was determined when *P* < 0.05. All the relative data was analyzed by the SPSS Statistical Software version 19.0 (SPSS Inc., Chicago, IL, USA).

## Results

### Literature search

**Fig. 1:**
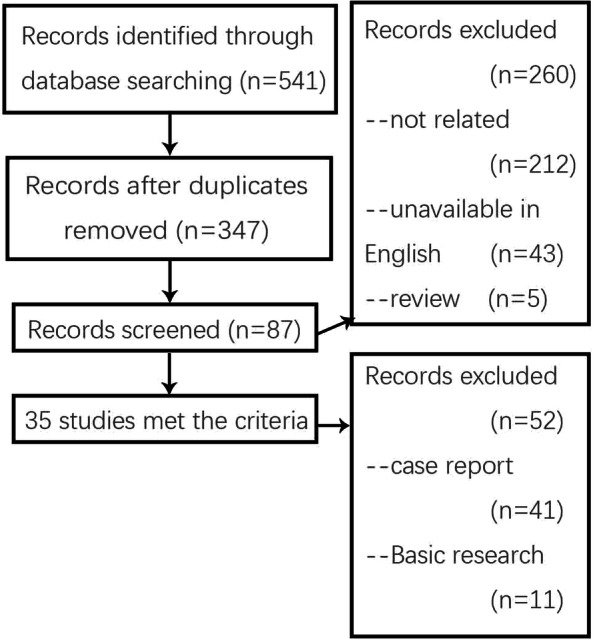
Flow chart illustrating number of studies evaluated at each stage in the systematic review

The initial literature search identified 541 records while 87 articles were identified as related articles on thoracic myelopathy. Ultimately, 35 studies met the inclusion criteria by reviewing the full-text articles (Fig. 1) which contained 5 retrospective comparative studies [6–10] and 30 case series [2, 4, 5, 11–37] (Table 1).

**Table 1:** Characteristics of included studies

Author	Study NO.	Number of patient	Gender ratio (male/female)	Mean age (years)	Mean follow-up duration (months)	Cause of TM	Comorbidity	Type of OPLL/OLF			Severe lesions		
								Single	Multiple but not fusion	Fusion	T1–T5	T5–T9	T9–TL1
Wang et al.	1	33	1.20	55.03	70.82	OLF	DM 2 + CM 2 + LM 11	16			9	4	52
Wang et al.	2	18	0.38	56.2	31.2	OLF	DO 11	5	0	13	1	6	33
Li et al.	3	85	0.57	60.2	49.2	OLF	DM 9				22	19	44
Hou et al.	4	427	1.42	53		OLF 309 + OPLL 120 + DH 129	CM 61 + LM 47						
Onishi et al.	5	73	1.70	61.9	70.2	OLF 55 + OPLL 25 + DH 8	DM16				11	18	44
Hitchon et al.	6	44	2.14	66	16		CM 13 + LM 6						
Uehara et al.	7	35	1.91	70.5	44	OLF 20 + OPLL 6 + DH 1					4	1	30
		15	1.50	63.5	46.1	OLF 5 + OPLL 4 + DH 3 + AS 3					0	1	14
Yang et al.	8	21	0.90	52.1	24.5	OPLL		11	3	7	27	11	3
Onishi et al.	9	15	0.87	65.5	45	OLF 15 + OPLL 15	DM 4				3	10	2
Wang et al.	10	23	0.84	39.8		OLF 99 + OPLL 66 + DH 17							
		145											
Li et al.	11	11	0.57	50.2	21.2	OLF 11 + OPLL 9	DO 8				19	22	24
He et al.	12	283	1.62	51.8		OLF 123 + OPLL 73 + DH 54 + DISH 19					49	60	174
Ma et al.	13	23	1.55	34.7	4.6		DO 5						
Kawaguchi et al.	14	41	3.10	59.4	66	OLF	DM 11						
Yang et al.	15	38	1.37	59	46.1	OLF		6	32			23	13
Nie et al.	16	18	0.80	56.3	35	OLF	DO 2		18				
Liu et al.	17	13	0.62	56	36.8	OLF 13 + OPLL 13	DO 2						
Li et al.	18	19	0.58	55.3	53.2	OLF	DM 2						
Yu et al.	19	78	2.25	59.6		OLF		63	15			22	
													15
Gao et al.	20	75	1.34	54.7	35.7	OLF	CM 12 + LM 7		75				
Li et al.	21	7	0.75	56.7	74.4	OL 31F + OPLL 31							
		11	0.83	56.8	52.8								
		13	0.44	57.3	90.2								
Sun et al.	22	266	1.37	54.3		OLF	DO 67						
Yamazaki et al.	23	24	0.41	54.8	53	OPLL							
Zhang et al.	24	11	2.66	54.5		OLF 11 + OPLL 11	CM 1						
Chen et al.	25	16	1.66	57.5	57.3	OLF	CM 1 + LM 2				4	4	21
Matsuyama et al.	26	37		58		OPLL							
Hur et al.	27	26	1.88	53	27.3	OLF		1	5	20	10	6	25
Zhong et al.	28	22	1.00	52.4	35.6	OLF	CM 4 + LM 3	3	8	11	2	1	14
Yoon et al.	29	40	1.85	57.08	17.3								
Park et al.	30	12	0.50	60.5	24.2	OLF		10	2				
Zhao et al.	31	13	1.16		13.3	OLF							
Zhang et al.	32	56	2.29		24	OLF	DM 23 + DO 48	33	23			4	3
		40	2.07					20	29			3	5
Hirabayashi et al.	33	13	3.33	58		OLF							
Takahata et al.	34	30	0.66	53	96	OLF	CM 15						
Khoo et al.	35	13	0.63	51.8	12	DH							

The blanks are not mentioned in the study

*DM* diabetes mellitus, *DP* dura ossification, *CM* cervical myelopathy, *LM* lumbar myelopathy

### Study details

A total of 2170 patients were included in our systematic review. 56.9% of patients were male, and 43.1% were female. The average age of patients was 55.2 years, and the mean follow-up ranged from 13.3 months to 96 months, and 43.0 months in total.

### Characteristics of thoracic myelopathy

Within all the patients, there were 69.8% patients diagnosed as OLF, 20.0% as OPLL, 9.3% as DH, and others including DISH and AS. There were some comorbidities in patients with thoracic myelopathy, including 18.5% with diabetes mellitus (67 in 362), 15.3% with cervical myelopathy (102 in 667), 19.9% with lumbar myelopathy (133 in 667), and 32.1% with ossified dura matter (143 in 445). In all of the OPLL and OLF, 40.0% were single lesion type (168 in 420), 47.9% were multiple but non-fusion type (201 in 420) and 12.1% were fusion type (51 in 420). Besides, in all spine stenosis lesions, upper thoracic lesions (T1–T4) represented 19.9% (213 in 1070), middle thoracic lesions (T5–T8) represented 18.6% (199 in 1070), and lower thoracic lesions (T9–L1) represented 61.5% (658 in 1070).

### Surgical procedures

We categorized all the surgical procedures for thoracic myelopathy into three major procedures: (1) posterior decompression, (2) circumferential decompression, and (3) minimally invasive surgery. However, it is a pity that only two study applied minimally invasive surgery on 26 patients suffered from single lesion type OLF, and the number of patients is too small to come up with a statistical difference between minimally invasive surgery and other surgery approach. Therefore, we mainly focused on the differences between PD and CD.

Mostly, surgeons determine surgical approach according to the compression location and etiologies. Causes of thoracic myelopathy and surgical approach were reported in 27 studies; there were 1102 OLF lesions treated by PD and 61 by CD and 222 OPLL lesions treated by PD and 52 by CD. Patients with disc herniation were all treated by PD. The statistical results showed that posterior decompression was more performed for OLF and circumferential decompression was more applied for OPLL (*p* < 0.01).

We found that the volume of blood loss was more in CD than PD (*p* < 0.01), and there was no statistical difference in vertebral fusion amount and operation time (*p* > 0.05). Predictably, the blood loss and operation time were both extremely low in minimally invasive surgery.

As for the complications, there were 231 cases in 1311 PD (17.62%) patients and 48 cases in 81 CD (59.26%) patients, where the dural tear and leakage of CSF were the major complications. The incidence of complications was lower in PD than CD (*p* < 0.01). Besides, it is remarkable that the incidence of immediately neurologic deterioration in CD was extremely higher than PD. Lastly, the incidence of complications in minimally invasive surgery was 19.8%.

### Outcomes of surgery

The mJOA score ranged from 3.14 to 7.03 before surgery and averaged in 5.27, while ranged from 4.60 to 9.52 after surgery and averaged in 7.80. Besides, the post-operation recovery rate ranged from 0.15 to 0.67 and averaged in 0.46. In the last follow-up, the mJOA score ranged from 6.63 to 9.52 and averaged in 8.18, while the recovery rate ranged from 0.24 to 0.69 and averaged in 0.51 (Table 2). When comparing the PD and CD, the average postoperation RR was 0.49 in PD and 0.35 in CD, while the average last follow-up RR was 0.54 in PD and 0.55 in CD, which mean that the short-term outcome of PD was better than CD while there was no statistical difference in long-term outcome (Table 3). Interestingly, we could find that surgeons chosen CD because of the poor mJOA score before surgery, which mean that a poor RR of CD is predictable after the surgery. As for the MIS, the RR was 0.29 after operation and 0.49 in the last follow-up. When focus on the OLF separately, incidence of complications in PD was much lower than CD, with less blood loss and the higher recovery rate (Table 4). In OPLL, incidence of complications in PD was much lower than CD with less blood loss, but there was no statistical difference in recovery rate. Although the blood loss and the incidence of complications in patients with minimally invasive surgery was comparatively low, the short-term outcome was unsatisfactory.

**Table 2:** Interventions and outcomes of included studies

Study NO.	Surgical procedure	Number of vertebral fusion	Blood loss (ml)	Operation time (min)	Complications					Pre-operation mJOA	Post-operation mJOA	Recovery rate	Last follow-up mJOA	Last follow-up recovery rate
					Dural tear	Leakage of CSF	Epidural hematoma	Immediate neurologic deterioration	Wound infection					
1	PD	9	206.67	123.94		4		2	2	7.03	9.52	0.63	9.52	0.63
2	PD		691.1	231.7						4.1	7.8	0.54	7.8	0.54
3	PD	28	495.3	143.5	17	9		9	3	3.8			8.2	0.61
4														
5	PD 66 + CD 7	12	269	257	9		2	3	2	6			7.7	0.34
6	PD + CD	23				3	1			6.8			8.3	0.36
7	PD 42 + CD 8		353.7	193		3		1		6.8	7.8	0.24	7.8	0.24
			284.8	148.7		1			1	5.2	7.7	0.43	7.7	0.43
8	CD	21	1619	240		5		7	1	4.5	7.4	0.45	7.8	0.51
9	PD 10 + CD 5	7	764	418	5	4		3		5.7			7	0.25
10	PD	66	810.2	193.9				23						
			749.9	201.5				0						
11	PD	11	2268	520.4		10	1		1	3.5	4.6	0.15	7.5	0.53
12	PD	283			11	5	1		2					
13	CD		1350	276		6		3		4.3	6.1	0.27	8.5	0.63
14	PD	14	570	230		10				4.2	6.2	0.29	6.8	0.38
15	PD		340			11				5.39			8.97	0.64
16	PD		462.8	189.4		4				4.7	7.9	0.51	8.8	0.65
17	PD	13	1035.4	274		3		1	1	4.3	7.2	0.43	8.5	0.63
18	PD		954	273	4					3.16	8.34	0.66	8.34	0.66
19	PD	29								5.77	8.68	0.56	8.68	0.56
										5.33	6.73	0.25	6.73	0.25
										5.68	8.54	0.54	8.54	0.54
20	PD									5.8	8.2	0.46	8.2	0.46
21	CD		1630	374		4				3.14	8.11	0.63	8.36	0.66
	PD	11	1284	326		2				3.23	8.42	0.67	8.57	0.69
	PD		1020	268		3		4	1	3.92	7.08	0.45	6.63	0.38
22	PD				85	85			2					
23	PD	24				1		1		3.7	6.4	0.37	8	0.59
24						2				3.5	6.8	0.44	8.5	0.67
25	PD				3	3	1			5			7.7	0.45
26	PD	37	926	450						6.2	8.9	0.56	8.9	0.56
27	PD	26			4	4			1	6.65			8.17	0.35
28	PD	10			5	1	1	1	1	5.64	9.18	0.66	9.18	0.66
29	PD	0			6					7.1			8.58	0.38
30	PD					4				4.9	7.9	0.49	7.9	0.49
31	MIS	0	19.77	98.23						4.15	6.15	0.29	7.54	0.49
32	PD	6				3	2		0	5.7	9.2	0.66	9.2	0.66
	PD	4				4	2		1	5.4	7.2	0.32	7.2	0.32
33	PD									5.2	7.6	0.41	7.6	0.41
34	CD	26	1883	389	12			10	1	3.4	5.5	0.28	7.1	0.49
35	MIS	0	33	93.75		1		4						

The blanks are not mentioned in the study

*CSF* cerebrospinal fluid

**Table 3:** Comparison of relative data among three surgical approaches

Surgical procedure	Epidural hematoma	Immediate neurologic deterioration	Wound infection	Incidence of complications	Pre-mJOA	post-mJOA	Post-recovery rate	Last mJOA	Last recovery rate
PD	6	6	6	17.62%	5.22	8.07	0.49	8.32	0.54
CD	0	20	1	59.26%	3.92	6.39	0.35	7.79	0.55
MIS	0	4	0	19.2%	4.15	6.15	0.29	7.54	0.49

**Table 4:** Comparison of PD and CD for patients with OLF or OPLL

	Patient amount	Incidence of complications	Blood loss (ml)	Operation time (min)	Pre-mJOA	Post-mJOA	Post-recovery rate	Last mJOA	Last recovery rate
PD for OLF	680	34.6%	492.5	179.8	5.40	8.34	0.525	8.41	0.538
CD for OLF	30	76.7%	1883	389	3.4	5.5	0.28	7.1	0.49
PD for OPLL	61	8.3%	926	450	5.22	7.92	0.467	8.54	0.574
CD for OPLL	21	61.9%	1619	240	4.5	7.4	0.45	7.8	0.51

## Discussion

In this systematic review, we analyzed the safety and effect of each surgical procedure for patients with thoracic myelopathy, and a number of characteristics on patients with thoracic myelopathy were provided in our study. Besides, the difference between PD and CD for OPLL or OLF was separately analyzed. To our best knowledge, this is the first systematic review to analyze the difference between PD and CD for patients with OPLL or OLF. Our results showed that PD was better than CD for patients with OLF according to the safety and effect of each procedures, and PD was safer than CD for patients with OPLL

In 35 retrospective case series, 56.9% patients were male and 43.1% were female which stated that thoracic myelopathy may prefer a male population, which was contrary to a systematic review about thoracic OPLL [38]. The inconsistence was mainly caused by different target patients, because the major cause of thoracic myelopathy was OLF (69.8%). Meanwhile, we found that the incidence of diabetes mellitus was 18.5% in thoracic myelopathy patients which was much higher than general population (8.3% in 2013). It stated that diabetes mellitus may be related with thoracic myelopathy somehow.

It was reported that local mechanical stress could accelerate the ossification procedure [39]. Chen et al. hypothesized that the unstable circumstance in spine myelopathy may cause an ossification of other lesion ligament [40]. In this systematic review, the coincidence of cervical and lumbar myelopathy was 15.3% and 19.9%, which was much less than the ratio in a study [6]. However, these statistics could not definitely support this hypothesis because the causality between thoracic myelopathy and cervical/lumbar myelopathy remains unclear. Dural ossification happened in 32.1% patients because severe OLF and OPLL often came up with adhesion between dura matter and ossified ligament. Lastly, thoracic myelopathy in low lesions (T9-L1) happened in major (61.5%).

In the past 10 years, the surgical technique of PD or CD was improved, so the technique was a little bit different among all studies. For example, there were laminectomy [12], laminoplasty, foraminotomy, and others called posterior decompression while there was a minor difference among them. In this systematic review, we categorized the surgical procedures for thoracic myelopathy into three: PD, CD, and MIS. Unfortunately, only 2 studies reported minimally invasive surgery and it only reported 26 cases, so we mainly compared the differences between PD and CD. Up to now, surgeons chose surgical procedures mainly according to the location of myelopathy.

It was reported that the indication of circumferential decompression is localized spinal cord compression by a large OPLL in the kyphotic curve [41]. However, there was not an indication applied by surgeons strictly. So the indication cannot be analyzed in this systematic review.

We found that the volume of blood loss was more in CD than PD (*p* < 0.01), and there was no statistical difference in vertebral fusion amount and operation time (*p* > 0.05). When considering the difference in blood loss, we could not ignore that CD is an extra anterior decompression compared to PD, so the incision was larger in CD than PD. Besides, the blood loss and operation time were both extremely low in MIS. The main reason for these was that the surgery is only managing a single lesion [4, 5]. The incidence of complications was 17.62% in PD, 59.26% in CD, and 19.2% in MIS. However, it was remarkable that the incidence of immediately neurologic deterioration in CD was extremely high. It may be anterior decompression or resection of adhesion between dura matter and ossified ligament through posterior approach often caused damage on the spine cord and nerve root [37]. To decrease the incidence of complications, some surgeons suggested a technique that we should keep a floating fragment adherent to the dura matter [26], but the kind of decompression was not satisfactory when compared with a complete decompression. There was another strategy that increasing the mean arterial pressure could prevent the neurologic deterioration, because Wang et al. found that mean arterial pressure less than 81 mmHg was one of risk factors [7]. Besides, to release the immediately neurologic deterioration, glucocorticoid was suggested to ease the inflammation after surgery.

As for the outcomes of surgery, postoperation recovery rate was 0.49 in PD, 0.35 in CD, and 0.29 in MIS while at last follow-up, the recovery rate was 0.54 in PD, 0.55 in CD, and 0.49 in MIS. Therefore, the short-term outcome of PD was much better than CD and MIS but there was no statistical difference on the long-term outcomes. It is remarkable that mJOA score of CD before surgery is poorer, which mean that surgeons preferred CD for severe thoracic myelopathy and this may result in a poorer recovery rate than PD. Indeed, the principle on treating spinal pathology is to approach the pathology as much as possible. However, in Li’s article, surgeons have chosen PD and CD for patients who had similar mJOA score before surgery while there was no difference on RR after surgery [9]. His results showed that PD has a same efficacy as CD for severe thoracic myelopathy. In summary, our results in the systematic review showed that PD was safer and better for patients with thoracic myelopathy than CD. Besides, the safety of MIS was satisfactory while the outcome was not, which might be because of the unaggressive surgeons.

We also analyzed the difference between PD and CD for patients with OPLL or OLF. According to the outcome from 20 articles, PD was safer than CD whether OLF or OPLL, and the outcome of surgery in PD was much better than CD for OLF but nearly same for OPLL. Therefore, when choosing the surgical procedures for patients with OPLL or OLF, PD was suggested. Because there was no such a study of DH independently and the morbidity of thoracic DH is much lower than OLF/OPLL, we did not analyze the difference between PD and CD for thoracic DH.

This systematic review has some limitations. Firstly, there was no prospective research and only 5 retrospective cohort research. Secondly, there were only 2 articles about MIS included and the number of patients was too poor to come up with a statistical significance. Thirdly, the severity of thoracic myelopathy was not discussed in articles included.

## Conclusion

This systematic review showed that posterior decompression for thoracic myelopathy is safer and better than circumferential decompression according to the complication rate and surgical outcome. And we should also consider the location of compression before the operation.

## Data Availability

All data used and analyzed during this study are available from the corresponding author upon reasonable request.

## Abbreviations

OLF: Ossification of ligamentum flavum
OPLL: Ossification of posterior longitudinal ligament
DH: Disk herniation
DISH: Diffuse idiopathic skeletal hyperostosis
AS: Ankylosing spondylitis
CD: Circumferential decompression
PD: Posterior decompression
MIS: Minimally invasive surgery
mJOA score: Modified Japanese Orthopedic Association Scoring System
